# Identification and Characterization of the Seed Storage Proteins and Related Genes of *Cannabis sativa* L.

**DOI:** 10.3389/fnut.2021.678421

**Published:** 2021-06-07

**Authors:** Xin Sun, Yao Sun, Yao Li, Qiong Wu, Lei Wang

**Affiliations:** Department of Biotechnology, Institute of Advanced Technology, Heilongjiang Academy of Sciences, Harbin, China

**Keywords:** *Cannabis sativa*, seed storage protein, albumin, edestin, vicilin-like protein

## Abstract

Hemp (*Cannabis sativa* L.) seed is emerging as a novel source of plant protein owing to its rich protein content and reasonable nutritional structure. In the current study, the storage proteins of hemp seed were extracted using different methods. The modified Osborne method yielded maximum extraction of the hemp seed storage proteins, while degreasing had little effect on the hemp seed protein (HSP) extraction. Protein identification results revealed that 11S globulin (edestin) was the most abundant protein in hemp seed, and the molecular weights of the two subunits of this protein were ~35 and 20 kDa, respectively. The second most abundant protein was 2S albumin (Cs2S), with a molecular weight of ~14–15 kDa. The least abundant protein was 7S vicilin-like protein (Cs7S), with a molecular weight of ~47 kDa. Subsequently, gene families encoding these three storage protein classes, including three genes for edestin, two for Cs2S, and one for Cs7S, were cloned and then analyzed for amino acid composition and structure. The three edestins were different in their amino acid sequences and calculated molecular weights. The analysis of coding sequences revealed a higher percentage of similarity (62.7%) between *Edestin1* and *Edestin3*, while the similarity decreased significantly to ~57% between *Edestin1* and *Edestin2*, and 58% between *Edestin2* and *Edestin3*. The calculated protein molecular weight was the highest for the protein encoded by *Edestin1* and the smallest for the protein encoded by *Edestin2*. All three edestins were rich in arginine, while Edestin3 had a higher methionine content relative to that in the other two, which proved that Edestin3 had a better nutritional value. Cs2S and Cs7S were different from those reported in previous studies. Therefore, it could be inferred that amino acid composition varies with different hemp cultivars. The current research brought significant theoretical advance in illuminating the understanding of hemp seed storage protein and would have significance for future research on improving the nutritional quality of hemp seed and developing bioactive peptides.

## Introduction

Hemp (*Cannabis sativa* L.) has been widely used as a fiber source for a long time, with the earliest history records from 8,000 BC when people used its fiber to make rope for pottery (1). Industrial hemp (*C. sativa* L. supsp. *sativa*), generally refer to hemp, has <0.3% of tetrahydrocannabinol (THC), a higher plant height, and the main parts used are seeds and fiber (2). Owing to its low THC content, industrial hemp is unable to stimulate human nerves, therefore, has a potential to be grown as a safe crop (3).

Besides fiber, hemp seeds, which commonly refer to seeds from industrial hemp, is a high-quality source of protein and oil (4). Hemp seeds contain ~20–25% protein, 25–35% oil, 20–30% carbohydrates, and 10% fiber (5, 6). The hemp seed protein (HSP) is mainly composed of three types of protein, namely, 11S globulin (edestin), 2S albumin, and 7S vicilin-like protein, which account for ~60–80%, 13%, and 5% of total seed protein, respectively (7–9). Research on nutritional composition of seeds from different hemp cultivars has proved that the contents of protein exhibit significant variations among different cultivars, and are influenced by agronomic conditions (temperature and total rainfall), and mainly genotype. Analysis of variance revealed that the genotypic variance accounted for 52.4% of the total variation in the protein content. The effect of growing year was also significant. It was also found that geography, climatic conditions, and local agronomic factors may affect hemp seed nutritional composition (10). The amount of protein in hemp seeds was higher than or similar to other protein-rich products, such as quinoa (13.0%), buckwheat seeds (27.8%), and linseeds (20.9%) (11, 12). Due to its high protein content, inclusion of hemp seed can increase the protein content of food products. It was reported that about 38% increase in protein content was observed when replacing up to 20% of wheat flour with hemp seed flour to make bread (13), hence, hemp seed can be considered a good protein source.

The nutritional quality of a protein is determined by its amino acid composition, digestibility, and bioavailability. HSP is rich in all 9 essential amino acids, in amounts that are sufficient for 2 to 5-year-old children as recommended by FAO/WHO (14, 15). The most abundant amino acid of HSP is glutamic acid (3.74–4.58%) followed by arginine (2.28–3.10%). Glutamate can be used as a neurotransmitter in brain, and although not an essential amino acid, arginine is a dietary precursor for the formation of nitric oxide (NO), therefore it is with beneficial cardiovascular properties and has been linked to optimal immune function and muscle repair (16). In addition, HSP provides high quantities of sulfur-containing amino acids (methionine and cystine), which may serve as a nutritional tool to boost the antioxidant capacity of human body (15). The proportion of essential amino acids to the total amino acids (E/T) for HSP was 45.16%, which significantly higher than that of soybean proteins (41.72%), suggesting that HSP has more nutritional amino acid pattern than that of soybean proteins (12). Furthermore, HSP does not contain trypsin inhibitor, which is considered one of the most important antinutritional factors and is found in many species of graminaceous, cruciferous and leguminosae, rendering the former easier to digest and absorb in the human body (3, 17, 18).

It was found that the solubility of HSP at acidic pH was lower than that of soy proteins, which may be due to the formation of covalent disulphide bonds among individual molecules of edestin, resulting in insoluble protein aggregates, it was also found that the denaturation temperature of HSP was 92°C (6, 19). Investigating on the *in vitro* digestibility of HSP showed that untreated HSP was much more digestible compared to soy proteins (14). Research on *in vivo* digestibility of HSP showed that it exhibited a high degree of digestibility compared to other plant protein sources, such as canola meal and borage meal, as they contained highly digestible amino acids (8, 20). Overall, nutritionally, the HSP is highly digestible and has a better amino acid composition compared to several other plant proteins. It can be considered a good viable, vegetable-based protein source for human diet (4). Although nutritional value of plant proteins is lightly lower than that of animal proteins, they have certain benefits (21–24). Specially, plant protein resources are more abundant, cheaper, and more accessible in relative that of animal proteins. In addition, the hydrolysates of plant protein have unique physiological functions (22, 25–29). After digestion with different proteases, the hydrolysates of HSP were demonstrated to exhibit various physiological activities *in vitro* as well as *in vivo*, with hemp proteins short-chain peptides such as WVYY and PSLPA exhibiting antioxidant ability, GVLY and LGV exhibiting anti-hypertension ability, and the hydrolysates exhibiting antiproliferative and hypocholesterolemic activities (30–36).

Acid-precipitation-alkali extraction method was used in most studies to extracted HSP, since it was easy to implement. However, protein denaturation is inevitable owing to the addition of a strong alkali during the extraction process, which may affect the results of the subsequent experiments. Osborne fractionation method can extract proteins of different solubility, because of its mild extraction conditions, it might be a better method for extracting HSP. In addition, there are commercial kits that claim can extract seed proteins gently and completely. In the present study, the two methods and protein extraction kit were used for extracting the hemp seed storage proteins, the extraction results of them were compared.

Although hemp seed storage proteins have been well-characterized at the biochemical level, the knowledge of gene families encoding these seed storage proteins and their structures remains to be limited. Amino acid composition is an important factor which determined nutritional quality of a protein. Proteins belonging to the same protein family may have different amino acid compositions, and the amino acid composition can be obtained from their gene sequences. Hence, study of genes encoding proteins is helpful to the study of the nutritional quality of proteins. Moreover, samples used in researches were mainly obtained from Europe and a few from Yunnan of China, and there is no detailed research on hemp plant samples from other regions, especially those in northern China. Cultivated species from different regions have different genotypes, which is the main factor that influences protein content, as described earlier. There is no research on the HSP structures from different regional varieties. The present study was aimed to fill this gap by extracting seed storage proteins from hemp plants grown in northern China, characterizing and analyzing the corresponding genes, which would bring significant theoretical advance in illuminating the understanding of nutritional quality of HSP and the study on its protein structure would be conducive to the identification of its active peptides. The present research would serve as a reference for the development of high-quality plant proteins and the application of active peptides in food and medicine.

## Materials and Methods

### Materials and Reagents

Fresh seeds of *C. sativa* var. Longma 5, which was a commercial variety from Heilongjiang Province, China (one of the major provinces in hemp cultivation), were obtained from Ms. Mu (General manager of Heilongjiang Heike Technology Co. Ltd). The chemicals including NaOH and NaCl were purchased from Hongyan Co. (Tianjin, China); NH_4_HCO_3_, acetonitrile, dithiothreitol, acetonitrile, formic acid, RNase-free DNase I and PBS buffer were purchased from Thermo Fisher Scientific (San Jose, CA, USA); n-hexane was purchased from Aladdin Co. (Shanghai, China); HCl was purchased from Kermel Co.(Tianjin, China); KOD enzyme was purchased from Toyobo Co. (Osaka, Japan); pGEM-T plasmid was purchased from Takara Co. (Kyoto, Japan); 5X protein loading buffer was purchased from Beyotime Co. (Shanghai, China); Coomassie brilliant blue R-250 was purchased from Solarbio Co. (Beijing, China). The kits including protein extraction kit and RNA extraction kit were purchased from Solarbio Co. (Beijing, China) and Tiangen Co. (Beijing, China), respectively. All reagents used in the study were of analytical grade or higher purity.

### Preparation of Hemp Protein Isolate (HPI)

The proteins were extracted from hemp seeds using the method of Tang with a few modifications (37). In the present work, both defatted and raw hemp seeds were used and three different methods were selected and compared to observe the effect of degreasing and different methods on the SDS results of protein extraction. First, the hemp seeds were ground to powder using liquid nitrogen, followed by dispersion of seed powder in n-hexane in a ratio of 1:4 (*w*/*v*) and then stirring for 3 h at room temperature. Next, the semi-defatted hemp flour was added into n-hexane in a ratio of 1:6 (*w*/*v*) and stirred overnight; after that, the hemp flour was put in fume hood to remove traces of n-hexane at room temperature. Subsequently, the defatted hemp flour was dissolved in deionized water in a ratio of 1:20 (*w*/*v*), the pH of the mixture was adjusted to 9.0–10.0 using 1 M NaOH, and then the mixture was stirred for 4 h at room temperature. The mixture was subjected to centrifugation at 8,000 *g* for 30 min at 20°C. The supernatant was recovered, its pH was adjusted to 5.0 using 1 M HCl, and after allowing it to stand for 10–20 min, the precipitate was collected through centrifugation at 8,000 *g* for 30 min at 20°C. The precipitate was washed three times with deionized water and resuspended in deionized water, with the pH adjusted to 7.0. The suspension was freeze-dried and stored in a refrigerator at −80°C until use.

The proteins from hemp seeds was also extracted using the protein extraction kit. Hemp seed powder was added to the RIPA (radio immunoprecipitation assay) lysis buffer (50 mM Tris (pH=7.4), 150 mM NaCl, 1% Nonidet P 40, 0.5% sodium deoxycholate) in a ratio of 1:5 (*w*/*v*), the mixture was stayed at 4°C for 20 min, followed by centrifugation at 13,000 g for 30 min at 4°C, the supernatants was freeze-dried and stored in a refrigerator at −80°C until use.

### Preparation of Albumin and Globulin From Hemp Seeds

**Figure 1:**
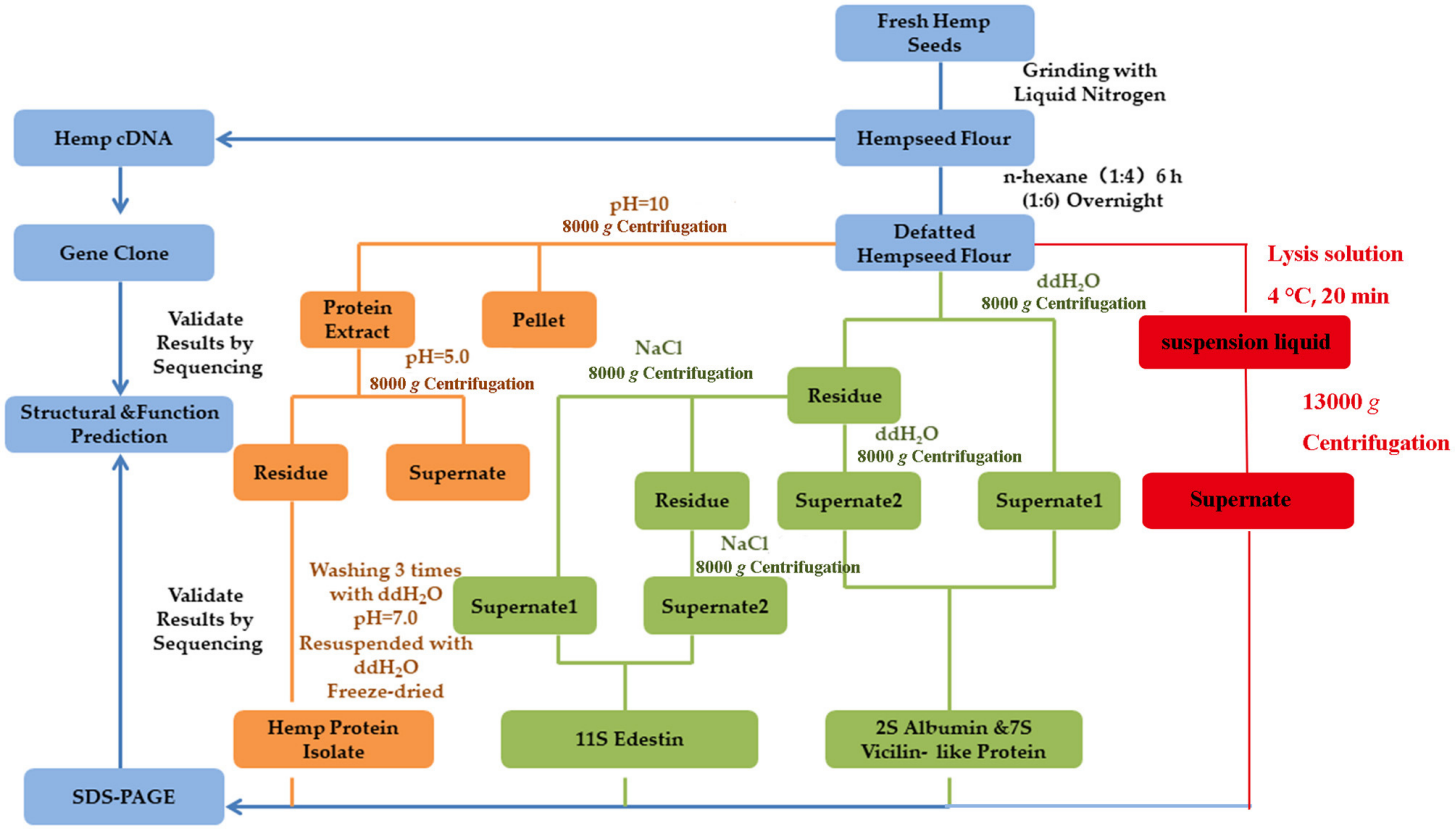
Flowchart of the production of HSP from fresh hemp seeds using different methods. Different colors indicate the different extraction methods; the Osborne method is indicated in GREEN, alkali extraction and acid precipitation method is indicated in YELLOW, protein extraction kit method is indicated in RED, and the common operating steps are indicated in BLUE. The concentration of NaOH and NaCl solutions used was 1 M, while the concentration of the HCl solution used was 0.1 M.

The isolation of 2S albumin, 11S globulin, and 7S vicilin-like protein present in the hemp seeds was performed using the Osborne fractionation method with slight modification. The degreasing method used for the isolation was the same as that described before. Hemp seed powder was added to deionized water in a ratio of 1:10 (*w*/*v*), and the mixture was stirred for 1 h at 37°C, followed by centrifugation at 8,000 *g* for 30 min at room temperature. The resultant supernatant was transferred to a clean tube, and the precipitate was added to deionized water in a ratio of 1:5 (*w*/*v*). The sample was stirred for 1 h at 37°C and centrifugated at 8,000 *g* for 20 min. The supernatants from two centrifugations were combined and used for obtaining the water-soluble protein (Cs2S). In order to obtain the salt-soluble proteins, 1 M NaCl solution was added to the precipitate in a ratio of 1:10 (*w*/*v*), followed by stirring for 1 h at 30°C and then centrifugation at 8,000 *g* for 20 min at room temperature; the supernatant was transferred to a clean tube. The same operation was repeated using the changed NaCl ratio of 1:5 (*w*/*v*), and the two supernatants were combined to obtain the salt-soluble proteins (11S globulin and 7S vicilin-like protein). The flowchart of production of HSP using different methods is presented in Figure 1.

### Sodium Dodecyl Sulfate-Polyacrylamide Gel Electrophoresis (SDS-PAGE) Analysis

The SDS-PAGE analysis was performed using 10% separating gel and 2% stacking gel. The samples were diluted to an appropriate concentration (1 mg/mL) using the PBS buffer, followed by the addition of 5X protein loading buffer containing β-mercaptoethanol, heating at 95°C for 5 min, and then cooling and centrifugation. Electrophoresis was performed at 80 V at room temperature in stacking gel, and afterward at 100 V, using Bio-rad Mini protein (Bio-rad, Hercules, USA). The composition of running buffer was 192 mM glycine, 25 mM Tris, and 0.1% SDS. After the completion of electrophoresis, the gels were stained with 0.25% Coomassie brilliant blue, decolored using 7% acetic acid [methanol:acetic:water = 1:1:8 (*v*/*v*/*v*)], and then retained for analysis. Different boiling times and 2ME (β-mercaptoethanol) contents were used as they may effect degree of protein denaturation.

### Protein Identification Analysis

The SDS-PAGE gel was soaked in 50 μL of pure water for 30 min and then removed and cut into 1–2 mm gel pieces, decolored using the decolorizing solution (50 mM NH_4_HCO_3_:acetonitrile 1:1, *v*/*v*) and dehydrated using acetonitrile until the micelles were completely white. This was followed by adding 10 mM DTT (dithiothreitol) to reduce the sulfhydryl groups, and then the micelles were dehydrated using 1 ug/μL trypsin and incubated overnight at 37°C. The next day, 5 volumes of 50% MeCN (acetonitrile) were added, and centrifugation was performed at 5,000 *g* for 1 min. The resulting supernatant was centrifuged at 25,000 g for 5 min, and the obtained supernatant was freeze-dried.

The dried peptide samples were reconstituted with mobile phase A [2% ACN, 0.1% FA (formic acid)], centrifuged at 20,000 *g* for 10 min, and the supernatant was taken for injection. Separation was carried out by a Shimadzu LC-20AD model nanoliter liquid chromatography system. The sample was first enriched in a trap column and desalted, and then entered a tandem self-packed C18 column (75 micron internal diameter, 3 micron column size, 20 cm column length) and separated at a flow rate of 300 nL/min by the following effective gradient: 0–8 min, 5% mobile phase B (98% ACN, 0.1% FA); 8–90 min, mobile phase B linearly increased from 8 to 21%; 90–102 min, mobile phase B rose from 21 to 32%; 102–110 min, mobile phase B increased from 32 to 80%; 110–115 min, 80% mobile phase B; 115–120 min, 5% mobile phase B. The nanoliter liquid phase separation end was directly connected to the mass spectrometer. The peptides separated using liquid-phase chromatography were ionized using a nanoESI source and then passed through a tandem mass spectrometer LTQ Orbitrap Velos (Thermo Fisher Scientific, San Jose, CA, USA) for DDA (data-dependent acquisition) mode detection. The main parameters were set: the ion source voltage was set to 2.2 kV; the MS1 scan range was 350–1,500 m/z; the resolution was set to 30,000; the MS2 starting m/z was fixed at 100; the resolution was 7,500. The screening conditions for the MS2 fragmentation were: charge 2+, 3+ and 4+ or higher, and the top 8 parent ions with the peak intensity exceeding 1,000. The ion fragmentation mode was HCD (higher energy C trap dissociation) with NCE (normalized collisional energy) set to 35, and the fragment ions were detected in Orbitrap. The dynamic exclusion time was set to 15 s.

Protein identification was achieved using experimental MS/MS data and aligning the data with the theoretical MS/MS data from a database to obtain the results. The databases used were the UniProt protein database and *Cannabis sativa* protein database in NCBI. The results from the search engine were pre-processed and re-scored using Percolator/[1/] to improve the matching accuracy. The output was then filtered at FDR 1% at the spectral level (PSM-level FDR ≤ 0.01) to obtain a significant identified spectrum and peptide list. Subsequently, based on the parsimony principle, protein inference was performed on the peptides to generate a series of protein groups. The peptide, protein and spectrum list of all the samples can been seen in the Supplementary Table 2.

### Cloning of Hemp Seed Storage Protein Gene Family

Fresh hemp seeds were ground with liquid nitrogen and subjected to total RNA isolation using the RNA extraction kit. The isolated total RNA was digested with RNase-free DNase I and then reverse-transcribed to be used as the template of PCR amplification. The RNA was detected using 2% agarose gel electrophoresis. The primers for gene cloning, presented in Supplementary Table 1, were obtained from E. Ponzoni's report (38). The PCR amplification was conducted using the KOD enzyme. The amplification program was as follows: 94°C for 5 min, 30 cycles of 15 s at 94°C, 30 s at 65°C, and 2.5 min at 68°C, and a final extension for 10 min at 68°C. The specific bands amplified in PCR were purification and cloned in the pGEM-T plasmid. The positive clones were sequenced in both directions.

### Sequence Analysis

All protein sequences obtained were compared with those of soybean proteins. Soybean proteins were selected because they are among the most important and commonly used plant sources of high-quality proteins. Sequences of soybean proteins were obtained from NCBI (https://www.ncbi.nlm.nih.gov/), accession numbers were P19594, P04347, and XP_003542001. Nucleotide sequences were analyzed using Condon code Aligner (https://www.codoncode.com/aligner/download.htm) and BLAST search programs in NCBI (https://blast.ncbi.nlm.nih.gov/Blast.cgi) to examine the sequences and identify their homologous genes. Pairwise sequence alignment and multiple alignments were performed using EMBOSS Needle and Clustal Omega (http://www.ebi.ac.uk/Tools). SignalP and NetNGlyc were employed to predict the putative N-terminal signal sequences and N-glycosylation sites, which were publicly available at (http://www.cbs.dtu.dk/services/SignalP/) and (http://www.cbs.dtu.dk/services/NetNGlyc/). The Phyre2, a website for predicting the secondary and tertiary protein structures, was used for predicting the structures of the concerned proteins (http://www.sbg.bio.ic.ac.uk/phyre2/html). Amino acid analysis and molecular mass estimation were performed using the ProtParam tool (https://web.expasy.org/protparam/).

## Results

### SDS-PAGE Analysis

**Figure 2:**
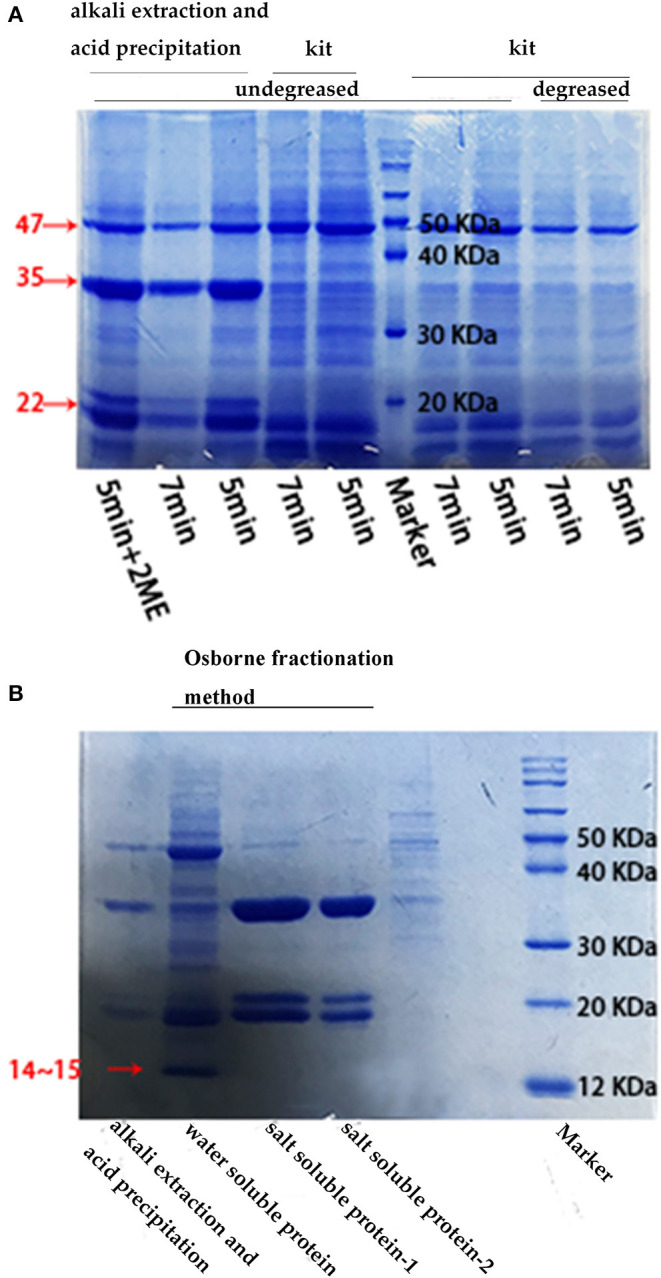
SDS-PAGE results under different conditions. **(A)** SDS-PAGE electrophoresis of defatted/undefatted hempseed protein extracted using different methods. The time indicated the protein boiling time at 95°C and the 2ME concentration was 2.5%; **(B)** SDS-PAGE electrophoresis of raw hempseed protein extracted using the “acid precipitation and alkaline extraction” method and the Osborne fractionation method (for water-soluble and salt-soluble proteins, respectively). The salt-soluble protein-1 was extracted using NaCl, while the salt-soluble protein-2 was extracted using urea. The concentrations of NaCl was 1 M and of urea was 8 M, the boiling time was 5 min.

The SDS-PAGE profiles of the proteins extracted using the different methods are presented in Figure 2. All proteins extracted using the different methods, except for the protein obtained using the kit method, contained three bands of sizes 47, 35, and 20 kDa. The proteins extracted using the kit had one band of size 47 kDa and two bands of size 18 kDa. The water-soluble proteins presented four bands of size 46, 35, 18, and 14 kDa, while the salt-soluble proteins also had four bands of size 47, 35, 22, and 18 kDa. The urea-soluble proteins exhibited the same pattern as that of the salt-soluble protein. As stated earlier, edestin has a hexamer structure composed of six identical subunits, each of which consists of an acidic subunit (AS) and a basic subunit (BS) linked via disulfide bonds. These disulfide bonds were broken during SDS-PAGE because of the presence of 2-ME, thereby generating the band of size 35 kDa for AS and bands of size around 20 kDa for BS. The 47 kDa band was for the 7S vicilin-like protein, while the minor water-soluble protein was probably albumin, as suggested in previous research.

The Osborne fractionation method appeared to be the best method for the extraction of hemp seed storage proteins as this method could extract all 3 types of proteins without denaturing the protein structures as no strong acid and alkali were used in this method. The acid-precipitation-alkali extraction method could extract most hemp seed storage proteins, although 2S albumin could not be obtained using this method; besides, the method may lead to protein denaturation because of the use of NaOH. The performance of the commercial plant protein extraction kit in the extraction of HSP was not quite satisfactory as it could extract only a small portion of the hemp seed storage protein. The proteins detected in the electrophoretic profiles were in the same range of molecular weight as that reported previously for these proteins (2S albumin: <15 kDa, edestin: 30 and 20 kDa, 7S vicilin-like protein: 47 kDa). In addition, degreasing, boiling time, and reducing agent content were observed to have little effect on hemp seed storage protein extraction and SDS-PAGE results.

### Protein Identification Analysis

The target bands of proteins were isolated using SDS-PAGE and then analyzed by LC-MS/MS. The databases used were UniProt protein database and *Cannabis sativa* protein database in NCBI. Using the search engine, results displayed that a total of 372 proteins and 1,521 peptides were identified from sample 40–50 kDa, 369 proteins and 1,333 peptides were identified from sample 35 kDa, 265 proteins and 773 peptides were identified from sample 20 kDa-1, 281 proteins and 797 peptides were identified from sample 20 kDa-2, and 126 proteins and 305 peptides were identified from sample <20 kDa using the search engine (Table 1).

**Table 1:** Identification results overview.

**Sample**	**Total spectra**	**Identified spectra**	**Identified peptides**	**Identified proteins**
40–50 kDa	12,877	2,461	1,521	372
35 kDa	12,483	2,992	1,333	369
20 kDa-1	12,524	2,842	773	265
20 kDa-2	11,835	3,119	797	281
<20 kDa	10,306	2,228	305	126

**Figure 3:**
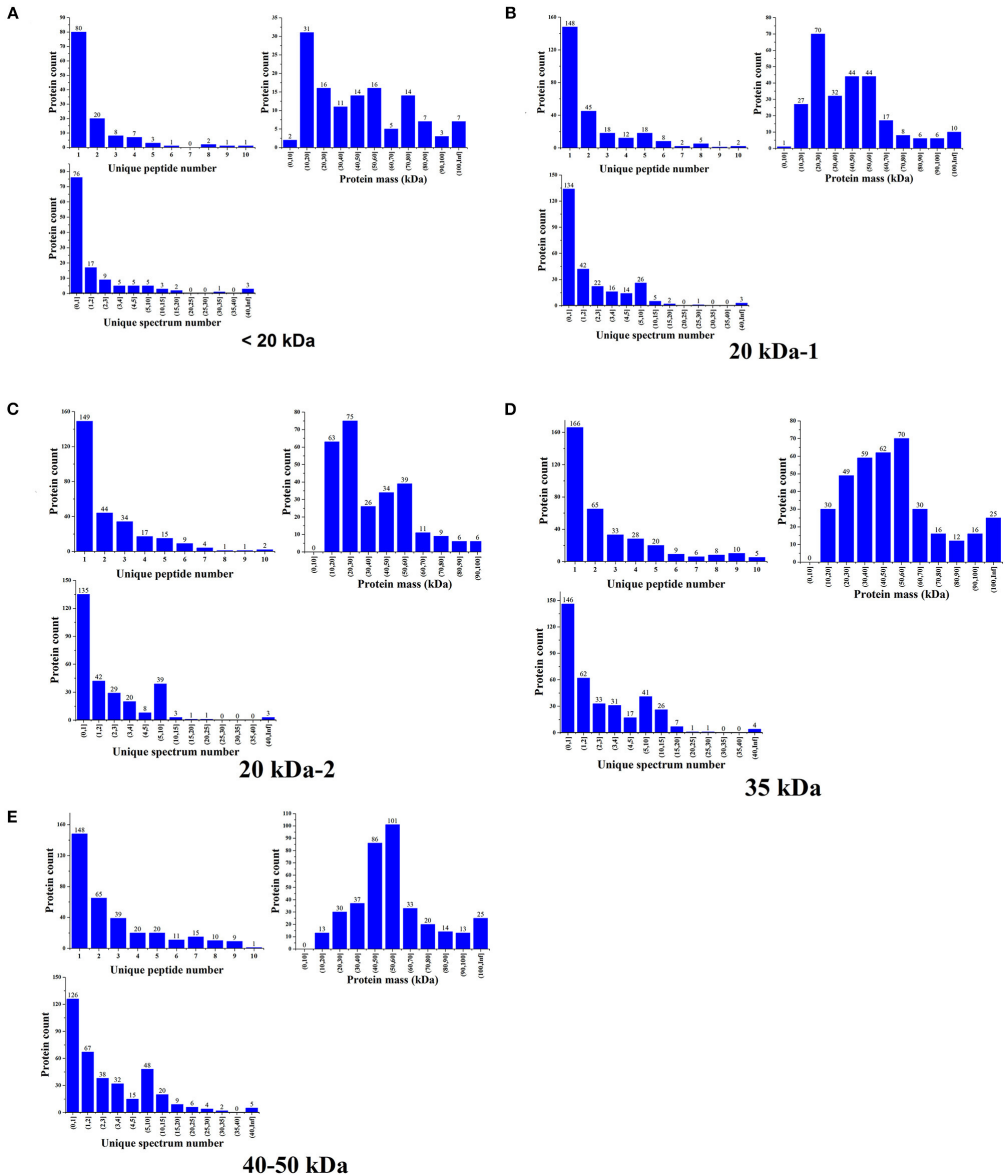
Basic statistical charts for the protein identifications. **(A)** Identification results for proteins in the 40–50 kDa range. **(B)** Identification results for proteins in the 30–40 kDa range. **(C,D)** Identification results for proteins in the 20 kDa range. **(E)** Identification results for proteins in the <20 kDa range.

The basic statistical charts for protein identifications were depicted in Figure 3. The results were filtered based on the Q score of identified proteins, numbers of unique peptides, coverage, and molecular weight, proteins with significant differences in molecular weight compared to those reported previously were excluded (data provided in Supplementary Table 2). It could be inferred that the identified protein from sample <20 kDa was Cs2S, that from samples 20 kDa-1, 20 kDa-2, and 35 kDa was edestin, and that from sample 40–50 kDa was Cs7S. It could be concluded from the identification results that the protein extracted was indeed the target protein. Three main types of seed storage proteins were present in this variety of hemp: Edestin, which was the most abundant seed storage protein and was composed of two subunits of molecular mass of 35 and 18–20 kDa, respectively; Cs2S, which had a molecular mass of 14 kDa; and Cs7S which was the least abundant and had a molecular mass of ~47 kDa.

### Cloning of the Gene Family Encoding Hemp Seed Storage Proteins

**Figure 4:**
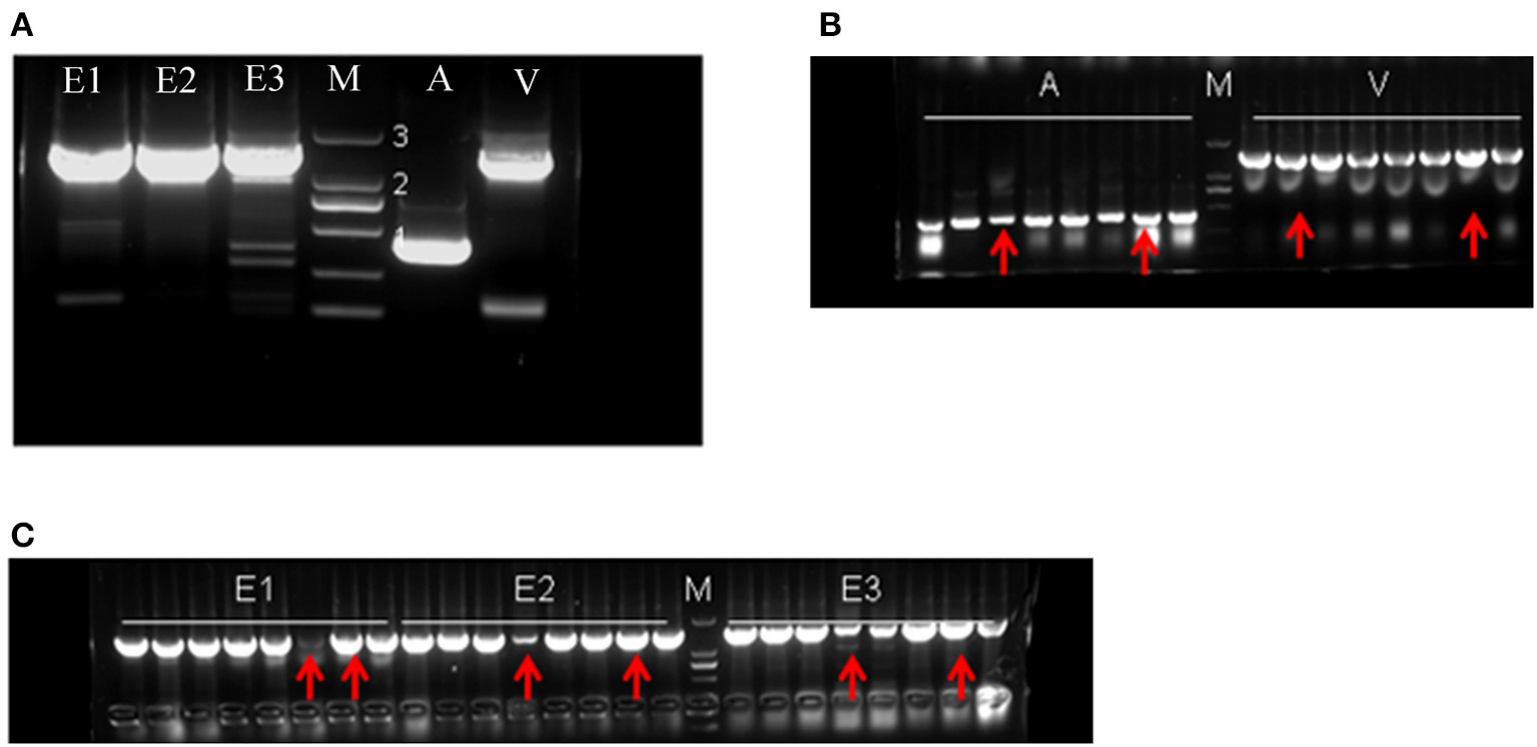
The electropherogram of gene cloning. **(A)** Result of PCR amplification. **(B,C)** PCR results for the recombinant vectors; the red arrows indicate the positive clones sent for sequencing. E1: edestin1; E2: edestin2; E3: edestin3; A: 2S albumin; V: 7S vicilin-like protein. Numbers 1, 2, and 3 indicate DNA marker bands, representing 500, 1,000, and 2,000 bp, respectively.

All target genes were amplified using PCR (Figure 4A). The genes encoding Edestin1, 2, and 3 were of 1,500 bp, the gene encoding Cs2S was of 400 bp, and the gene encoding Cs7S had ~1,500 bp. After purification, the target fragments were cloned into the T-vector, and the selected positive clones were subjected to sequencing (Figures 4B,C). A BLAST search of all putative genes in the NCBI database revealed a high identity and similarity (98%) with the hemp seed storage proteins (Supplementary Figure 1).

### Sequence Analysis of Hemp Seed Storage Protein Genes

#### 2S Albumin

The coding sequence of *Cs2S* obtained from hemp seed was 423 bp in length, encoding 140 amino acid residues with two glycosylation sites. This sequence also contained an N-terminal signal sequence comprising 23 residues for ER-targeting and a processing site for splitting into a small subunit and a large subunit.

The predicted molecular weight of Cs2S was 16.34 kDa, while the theoretical PI was 6.51 (determined using ExPASy compute pI/MV tool). The total number of negatively charged residues (Asp + Glu) was 20, and positively charged residues (Arg + Lys) was 19. BLAST results confirmed that it was indeed hemp 2S albumin. The secondary structure prediction data displayed that Cs2S had 70% helix and 11% TM helix content and no beta strands, indicating that the protein might have a transmembrane domain, which was in accordance with the SMART predicted results, Cs2S indeed had a transmembrane domain (7–24 amino acid residues) and a conservative AAI domain (48–135 amino acid residues). The AAI domain was represented by a structural domain consisting of 4 helices in a folded-leaf topology, forming a right-handed superhelix. Previous studies reported that this kind of domain occurred in several proteins, including the plant lipid transfer protein, seed storage proteins, and trypsin-alpha amylase inhibitor domain family of proteins.

**Figure 5:**
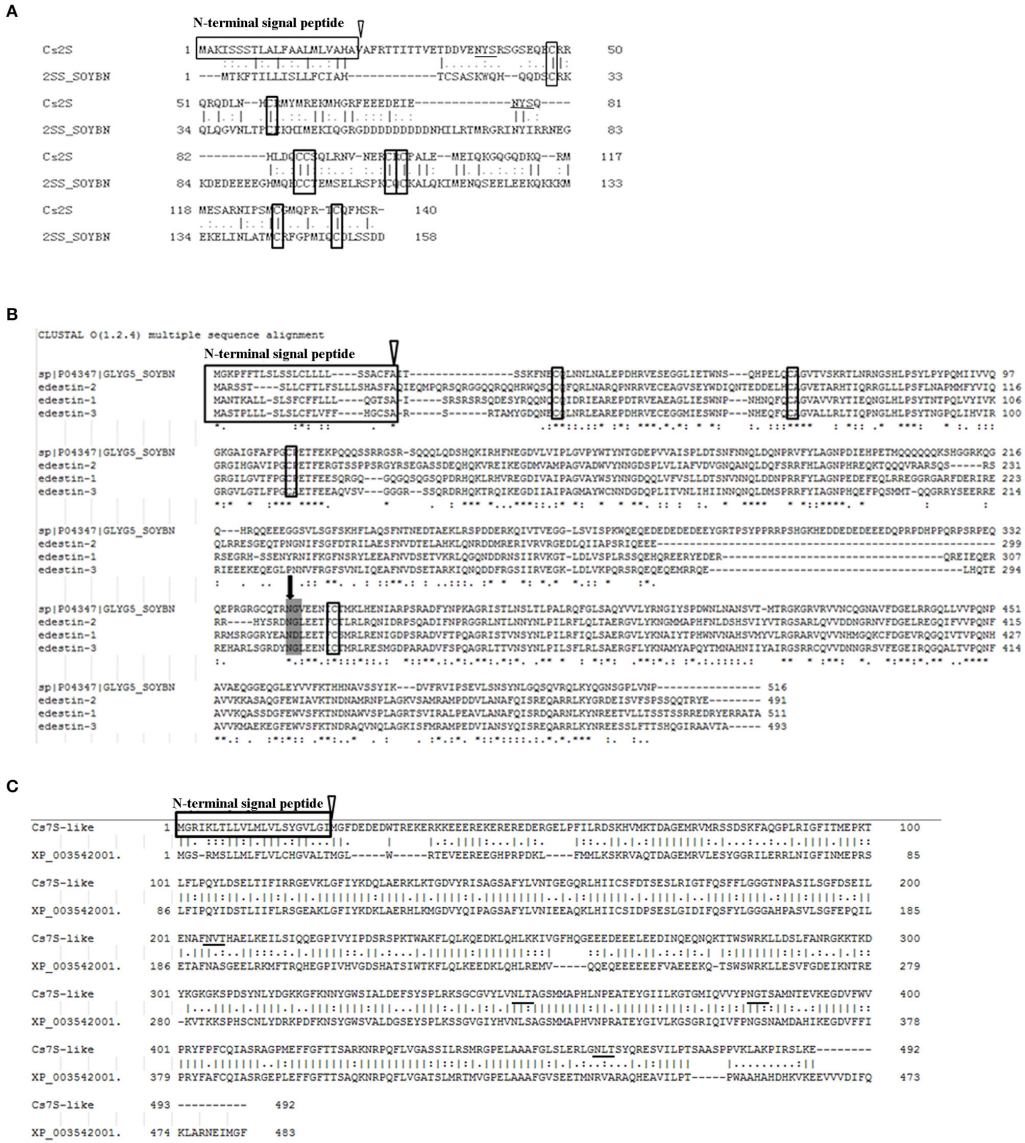
Alignment results of the coding sequences. **(A)** Alignment result for Cs2S and soybean 2S albumin. **(B)** Alignment results for the three Edestins and soybean 11S globulin. Broken lines were introduced to maximize similarity. Asterisks indicate identical amino acids. **(C)** Alignment result of Cs7S and soybean 7S vicilin-like protein (XP_003542001). The cleavage site of signal peptide is indicated by open triangle, while the conserved cysteine residues are boxed. The black arrows indicate cleavage site for splitting these polypeptides into an acidic subunit and a basic subunit. The underlines indicate the predicted glycosylation sites.

The alignment of Cs2S and soybean 2S albumin did not reveal a close association between these in the coding sequence. Cs2S was only 21.7% identical to the soybean 2S albumin, although both proteins had typical characteristics of 2S albumin, i.e., the N-terminal signal peptide targeting ER and 8 conserved cysteine residues linked via disulfide bonds (Figure 5A). The amino acid analysis revealed that soybean 2S albumin contained 20 amino acids, while Cs2S contained one less, which was tryptophan. In addition, both 2S albumins contained 9 essential amino acids required by human body. The most abundant amino acid in Cs2S was arginine, while that in soybean 2S albumin was Glu. Moreover, Cs2S contained higher contents of Val, Tyr, Arg, and Ala compared to soybean 2S albumin, while soybean 2S albumin contained more Lys than Cs2S. The E/T value of Cs2S was 34.3%, lower than soybean 2S albumin (41.1%), however, its content of sulfur-containing amino acids was higher than that of soybean (Table 2). The Arg/Lys ratio of Cs2S (3.74) was significantly higher than that of soybean (0.6). The tertiary structure prediction results revealed that most similar structure to Cs2S was the Brazil nut 2S albumin bere 1 (Supplementary Figure 2), which was composed of two large subunits and one small subunit connected via disulfide bonds.

**Table 2:** Amino acid composition of 2S albumin and 7S vicilin protein.

**Amino acid**	**Number**	**Proportion (%)**	**Number**	**Proportion (%)**	**Number**	**Proportion (%)**	**Number**	**Proportion (%)**
	**Cs2S**		**Soybean 2S**		**Cs7S**		**Soybean 7S**	
Ala(A)	9	6.428571	4	2.531646	25	5.081301	34	7.039337
Cys(C)^#^	8	5.714286	10	6.329114	3	0.609756	4	0.828157
Asp(D)	6	4.285714	15	9.493671	22	4.471545	17	3.519669
Glu(E)	14	10	17	**10.75949**	45	9.146341	47	**9.730849**
Phe(F)^*^	4	2.857143	3	1.898734	27	5.487805	28	5.797101
Gly(G)	5	3.571429	7	4.43038	42	8.536585	37	7.660455
His(H)^*^	5	3.571429	5	3.164557	6	1.219512	17	3.519669
Ile(I)^*^	5	3.571429	11	6.962025	28	5.691057	29	6.004141
Lys(K)^*^	4	2.857143	15	9.493671	38	7.723577	27	5.590062
Leu(L)^*^	8	5.714286	13	8.227848	50	**10.1626**	42	8.695652
Met(M)^*^^#^	10	7.142857	9	5.696203	13	2.642276	18	3.726708
Asn(N)	6	4.285714	6	3.797468	16	3.252033	14	2.898551
Pro(P)	3	2.142857	3	1.898734	22	4.471545	21	4.347826
Gln(Q)	12	8.571429	12	7.594937	18	3.658537	19	3.933747
Arg(R)	15	**10.71429**	9	5.696203	30	6.097561	27	5.590062
Ser(S)	11	7.857143	9	5.696203	39	7.926829	36	7.453416
Thr(T)^*^	7	5	7	4.43038	25	5.081301	18	3.726708
Val(V)^*^	5	3.571429	1	0.632911	21	4.268293	30	6.21118
Trp(W)^*^	0	0	1	0.632911	6	1.219512	6	1.242236
Tyr(Y)	3	2.142857	1	0.632911	16	3.252033	12	2.484472

*Amino acid number and proportin of 2S albumin and 7S vicilin-like protein in hemp and soybean. Cs2S: 2S albumin from hemp, soybean 2S: from NCBI, Cs7S: 7S vicilin-like protein from hemp, soybean 7S: GLy from NCBI. Bold indicated the most abundant amino acid*.

* *Indicated the essential amino acids*.

# *Indicated the sulfur-containing amino acids*.

#### 11S Globulin

Edestin1, Edestin2, and Edestin3 are all 11S globulins. Results of glycosylation site prediction and amino acid analysis revealed that all three edestins lacked a glycosylation site. Further, the number of amino acids in them was similar, where *Edestin1* encoding 511 amino acids, *Edestin2* encoding 491 amino acids, and *Edestin3* encoding 493 amino acids.

The DNA coding sequence alignment of the three edestins (using Clustal omega for amino acid sequence alignment) revealed significant differences between them. The similarity between *Edestin1* and *Edestin2* was only 57.8%, between *Edestin2* and *Edestin3* was 58%, between *Edestin1* and *Edestin3* was 62.7%. The results of the alignment of amino acid sequences were similar to those obtained for the alignment of cDNA sequences. The identity and similarity of *Edestin1* with *Edestin3* were higher than those with *Edestin2*, with 57.5 and 73.5% (Table 3).

**Table 3:** Percentage of sequence similarity of coding sequences (CDS), amino acid sequences of edestins.

**Sequence pairs**	**Coding sequence**		**Amino acid sequence**	
	**Identity (%)**	**Similarity(%)**	**Identity (%)**	**Similarity(%)**
Edestin1/Edestin2	57.8	57.8	47.2	64.1
Edestin1/Edestin3	62.7	62.7	57.5	73.5
Edestin2/Edestin3	58.0	58.0	47.6	64.3

The predicted molecular weight of Edestin1 was 58.56 kDa and the theoretical PI was 8.44. The total number of negatively charged residues in Edestin1 was 66 and that of positively charged residues was 69. The corresponding values for Edestin2 were 55.98 kDa, 9.02, 57, and 63, and for Edestin3 were 56.26 kDa, 7.63, 59, and 60 (Table 4). The secondary structure prediction results demonstrated that the three edestins had similar secondary structures, containing a transmembrane helix with ~30% beta strands and 20% alpha helix. Eedestin1 contained 20% alpha helix, 28% beta strands, and 3% TM helix. Edestin2 had 19% alpha helix, 31% beta strands, and 3% TM helix. Edestin3 had 18% alpha helix, 29% beta strands, and 3% TM helix. In the tertiary structure prediction, the most similar structure to these three edestins was that of the *Wrightia tinctoria* reveals auxin binding site 11S globulin (Supplementary Figure 2).

**Table 4:** Sequence length, protein mass, and submit mass of three edestins.

**Edestin type**	**Coding sequence length (bp)**	**Amino acid residues**	**Protein mass (kDa)**	**A-subunit mass (kDa)**	**B-subunit mass (kDa)**
Edestin-1	1,536	511	58.56	34.35	21.77
Edestin-2	1,476	491	55.98	32.58	20.84
Edestin-3	1,482	493	56.26	32.68	21.17

*The protein mass and subunit mass were calculated from ExPASy compute pI/Mw tool. A-subunit sequence is from the end of signal peptide to the cleavage site, B-subunit sequence is from the cleavage site to the end of protein amino acid sequence*.

The amino acid sequences of the three edestins were compared with those of soybean 11S globulin (GL5) (Figure 5B). All edestins had common features of 11S globulin, although there were a few differences among them. They also had an N-terminal signal peptide targeting ER, a cleavage site capable of splitting into two subunits (acidic subunit and basic subunit), four conservative cysteine residues, and two conservative Cupin structure domains. The Cupin family represented the conserved barrel domain of the “cupin” superfamily (“cupa” is the Latin term for a small barrel) and contained the 11S and 7S plant seed storage proteins and germins.

According to the amino acid composition analysis, 11S globulin, whether from hemp or soybean, contained 9 essential amino acids required by human body. The amino acid compositions of the three edestins were almost similar to each other, with Arg as the most abundant amino acid, while the most abundant amino acid in GL5 was Gln. Ala, Met, and Arg contents in the edestins were significantly higher than those in GL5, while GL5 had higher Pro content than that in the edestins. The content of Met was significantly lower in Edestin2 than that in the other two edestins and accounted for only 30% of the content in each of the other two edestins, while Edestin3 had more Cys and Met than those in the other two edestins. The E/T values and content of sulfur-containing amino acids of Edestin2 (34.2%) and Edestin3 (35.1%) were higher than those of GL5 (33.7%), while Edestin1 (32.7%) had a similar E/T value and content of sulfur-containing amino acids compared with GL5. The Arg/Lys of all the three edestins were 5.27, 5.32 and 4.00, which were higher than that of GL5 (1.74) (Table 5).

**Table 5:** Amino acid composition of 11S globulins.

**Amino acid**	**Number**	**Proportion (%)**	**Number**	**Proportion (%)**	**Number**	**Proportion (%)**	**Number**	**Proportion (%)**
	**E1**		**E2**		**E3**			**Soybean**
Ala(A)	32	6.2622309	35	7.1283096	34	6.8965517	20	3.875969
Cys(C)^#^	6	1.1741683	6	1.2219959	9	1.8255578	8	1.550388
Asp(D)	24	4.6966732	25	5.0916497	17	3.4482759	24	4.651163
Glu(E)	42	8.2191781	32	6.5173116	42	8.5192698	42	8.139535
Phe(F)^*^	20	3.9138943	23	4.6843177	21	4.2596349	18	3.488372
Gly(G)	34	6.6536204	33	6.7209776	35	7.0993915	41	7.945736
His(H)^*^	10	1.9569472	12	2.4439919	12	2.4340771	15	2.906977
Ile(I)^*^	22	4.3052838	22	4.4806517	28	5.6795132	17	3.294574
Lys(K)^*^	11	2.1526419	10	2.0366599	12	2.4340771	19	3.682171
Leu(L)^*^	36	7.0450098	37	7.5356415	37	7.505071	41	7.945736
Met(M)^*^^#^	5	0.9784736	12	2.4439919	15	3.0425963	5	0.968992
Asn(N)	34	6.6536204	29	5.9063136	32	6.4908722	33	6.395349
Pro(P)	18	3.5225049	21	4.2769857	20	4.0567951	38	7.364341
Gln(Q)	37	7.2407045	42	8.5539715	38	7.7079108	45	**8.72093**
Arg(R)	58	**11.350294**	53	**10.794297**	48	**9.7363083**	33	6.395349
Ser(S)	42	8.2191781	37	7.5356415	32	6.4908722	43	8.333333
Thr(T)^*^	22	4.3052838	18	3.6659878	20	4.0567951	21	4.069767
Val(V)^*^	36	7.0450098	30	6.1099796	25	5.0709939	34	6.589147
Trp(W)^*^	5	0.9784736	4	0.814664	3	0.6085193	4	0.775194
Tyr(Y)	17	3.3268102	10	2.0366599	13	2.6369168	15	2.906977

*Amino acid number and proportin of three different edestins. E1: Edestin type 1; E2: Edestin type 2; E3: Edestin type 3;Soybean: Glycinin G5 from NCBI, Bold indicated the most abundant amino acid*.

* *Indicated the essential amino acids*.

# *Indicated the sulfur-containing amino acids*.

In addition to the differences in amino acid composition, the proteins coded by these three edestin genes were different in molecular mass and subunits. The predicted molecular weight of Edestin1 was 58.56 kDa, that of its a-subunit was 34.35 kDa, and that of its b-subunit was 21.77 kDa, while the corresponding values for Edestin2 were 55.98, 32.58, and 20.84 kDa, and for Edestin3 were 56.26, 32.68, and 21.17 kDa. It could be observed that the molecular weight of the protein unit encoded by the different edestin genes was ~55–59 kDa in size, with the size of its a-subunit ~32–35 kDa and size of its b-subunit ~20–22 kDa. The molecular weight of the protein encoded by Edestin1 gene was higher than those encoded by the other two genes, with Edestin2 gene encoding the smallest edestin (Table 4). The molecular weight of the protein containing the N-terminal signal peptide and a-subunit sequence was calculated from the cleavage site of signal peptide to the cleavage site of acid–base subunit, while that for the b-subunit sequence was calculated from the cleavage site of acid–base subunit to the end of protein.

#### 7S Vicilin-Like Protein

The cDNA sequence of *Cs7S* was 1,479 bp in length, encoding 492 amino acids, its predicted molecular weight was 55.7 kDa and the theoretical PI was 7.63. The BLAST results from NCBI also confirmed that the protein encoded by the gene was hemp 7S vicilin-like protein. The secondary structure prediction results revealed that the protein encoded by Cs7S had 29% beta strands, 20% helix, and 3% TM helix. Similarly, as a secretory protein, Cs7S had one N-terminal signal peptide, one transmembrane region (7–25 amino acid residues), two coiled-coil regions (26–57 and 258–285 amino acid residues), and two Cupin conserved domains (58–212 and 311–460 amino acid residues), which were the typical domains of seed 7S and 11S storage proteins. The amino acid sequence alignment of Cs7S and Gly7S (7S vicilin protein from soybean) was conducted using EMBOSS Needle, which revealed the identity and similarity of 55.3 and 70.4%, respectively, between Cs7S and Gly7S (Figure 5).

Different from the 11S globulin and 2S albumin proteins from hemp seed, the 7S vicilin-like protein contained only one conserved cysteine residue, although it had four NXT glycosylation sites. The amino acid composition analysis revealed that Leu was the most abundant amino acid in the Cs7S protein, while Glu was the most abundant amino acid in Gly7S. The His content in Cs7S was significantly lower than that in the soybean protein. The E/T values of Cs7S (43.5%) and Gly7S (44.5%) were similar, however, Gly7S had more sulfur-containing amino acids (Table 2). The tertiary structure prediction results revealed that the protein with the most similar structure to Cs7S was vicilin from *Solanum melongena* (Supplementary Figure 2).

## Discussion

In the present research, hemp seed storage proteins were extracted using different methods, and the results were compared. The Osborne fractionation method used in the present study could result in different hemp seed storage proteins with solubility differences and would not lead to protein denaturation because of its mild extraction conditions, which was conducive to the subsequent experiments. The acid precipitation and alkali extraction method was the most common method for extracting HSP (39). Protein denaturation is inevitable when using these methods owing to the addition of a strong alkali during the extraction process, which may affect the results of the subsequent experiments. Therefore, the modified Osborne fractionation method might be a better extraction method for use in HSP research. Moreover, it was observed that degreasing had little effect on protein extraction results. The extracted proteins were qualitative analyzed and protein sequence alignment analyzed using LC-MS/MS and BLAST, respectively, which finally confirmed that the extracted proteins were indeed hemp seed storage proteins. In Longma 5, the main seed storage proteins were 11S globulin, 2S albumin, and 7S vicilin-like protein, among which 11S globulin was the most abundant, followed by 2S albumin and 7S vicilin-like protein.

Gene families encoding hemp seed storage proteins were cloned, followed by the analysis of the coding sequences and amino acid composition and the prediction of the secondary and tertiary structures of proteins. One 2S albumin gene was cloned in this research, the cDNA length of *Cs2S* was 423 bp, encoding 140 amino acids. The predicted molecular weight of Cs2S was 16.34 kDa, amino acid sequence analysis showed, in agreement with previous studies, that Cs2S had the typical structure of 2S albumin, an ER-binding signal sequence containing 23 amino acid residues at the N-terminal, eight conserved cysteine residues that form two intrachain disulfide bonds in the large subunit and two interchain disulfide bond linking subunits in the mature protein, and a cleavage site that divides the protein into two subunits (38, 40). The most abundant amino acid of Cs2S was Arg (10.7%), in comparison with soybean 2S albumin, Cs2S contained higher Val, Tyr, Arg, and Ala amino acid contents. Although the E/T value of Cs2S was lower than that of soybean 2S albumin, however, it has a higher content of sulfur-containing amino acids. This sulfur-rich proteins have no inhibitory activity against trypsin and could serve as a rich thiol source to formulate highly nutritious foods, since various plant food proteins, especially legumin proteins from soybean, pea, and beans, are deficient in sulfur. These results were consistent with those reported by Ponzoni et al. (38), which reported that 18% wt (weight) of Cs2S was due to the sulfur-containing amino acids, while they were different from the findings reported by McKernan et al. (41), with the latter reporting that albumin encoded 151 amino acids and contained higher Tyr and Pro and less Glu compared to the values obtained in the present work. The E/T value of Cs2S in the present work was higher than that in McKernan's research, which indicated that Longma 5 had a better amino acid composition.

The analyses identified three edestin genes, namely *Edestin1,2* and *3*. The predicted molecular weight of protein encoded by *Edestin1* was higher than that of proteins encoded by the other two genes, with *Edestin2* being the smallest. Edestin is a hexameric structure comprising six identical subunits, each of which consisted of an acidic subunit (AS) and a basic subunit (BS) linked by disulfide bonds. The molecular weight of AS was ~35 kDa and BS contained two subunits of ~20 kDa. The result was consisted with previous research from Svedberg and Stamm, which reported that AS was 34.0 kDa, while BS were 20.0 and 18.0 kDa (32). All three edestins had a typical structure of 11S globulin,which was consist with Ponzoni's research that all the edestins had an ER-binding signal peptide sequence containing 23 amino acid residues at the N-terminal and four conserved cysteine residues (38). The identity and similarity of the coding sequence of *Edestin1* with that of *Edestin3* were higher than those with *Edestin2*. Arginine was the most abundant amino acid in all the three edestins. Arginine accounts for ~11% of edestins when compared with <7% for most other food proteins, including the proteins from potato, wheat, maize, rice, soy (1). Arginine can enhance blood flow and contribute to the maintenance of normal blood pressure (42). Lysine and tryptophan are the main limiting amino acids in hempseed (5). The Arg/Lys ratio is a determinant of the cholesterolemic and antherogenic effects of a protein (43). The Arg/Lys ratio of edestins obtained in the present research were 5.27, 5.32 and 4.00, which were remarkably higher than that of soybean (1.41) or casein (0.46), suggesting strong potential for edestins utilization in the formulation of cardiovascular health-promoting food products. Hence, edestins can be considered as a nutritional and bioactive ingredient for the formulation of foods that promote cardiovascular health. It is noteworthy that the Met content of Edestin3 was significantly higher than that of the other two edestins, and it is well-recognized the Met content is related to the nutritional value of crops. Therefore, it could be inferred that Edestin3 had a better nutritional value than the other edestins.

There was one *Cs7S* cloned in the present work. The coding sequence of the *Cs7S* was 1,479 bp and encoded 492 amino acids, it had four N(X)T N-glycosylation sites (X represents any amino acid except proline), two conserved Cupin domains, and an N-terminal signal peptide, which are present in most of the secreted proteins. The result obtained for Cs7S was consistent with those reported by Ponzoni and Docimo, although the amino acid sequences reported were not exactly the same (38, 44). The most abundant amino acid of Cs7S was Leu (10.16%), the E/T value of it was 43.49, which was higher than albumin and edestins. However, comparing with albumin and edestins, Cs7S had a lower content of sulfur-containing amino acids and a lower Arg/Lys ratio. It can be seen that Cs7S has a good amino acid composition but is otherwise inferior to albumin and edestins.

Results obtained for Cs7S were slightly different from those reported by McKernan, who reported that potential Cs7S encoded 791 amino acids and contained higher Glu content than that observed for Cs7S in the present work (41). The differences in albumin and Cs7S proteins could be related to the plant genotype and growth areas. The hemp cultivar used in Ponzoni's research was Futura, which originated in France and was grown in Italy, and had a THC content of ~0.3%. The cultivar used in McKernan's research was Jamaican Lion which originated in Jamaica and had a THC content of ~9%, although it belongs to subspecies sativa. The hemp cultivar used in the present work was Longma 5, which originated in North China and has a THC content lower than 0.3%. According to the results, the genotypes of Futura and Longma 5 might be more similar compared to Jamaican Lion, as the former two have similar THC contents and protein amino acid composition. The seed storage proteins of Longma 5 and Futura have better nutrition value than those of Jamaican Lion, as the former two have a higher ratio of essential amino acids to total amino acids. Therefore, it was inferred that amino acid composition of seed storage proteins varies with the different cultivars of hemp. As stated earlier, the amino acid composition is an important factor affecting the bioactivities and the function of peptides (10), and it could be that the peptides from different hemp cultivars would exhibit different bioactivities and functions. Therefore, it is of great significance to study the different hemp cultivars for developing better bioactive peptides.

The dietary requirements of humans are not for protein *per se*, but for specific amounts of indispensable or essential amino acids (11). HSP provides all 9 essential amino acids with a balanced amino acid profile, which can be used not only as a nutritive additive but also as a functional ingredient in formulated foods to enhance the product quality attributes. Compared with other plant proteins, HSP has a lower allergenicity, which can be seen as a substitute for other proteins in some food products. Hence, HSP is rising in demand as an alternative source of plant protein. Moreover, HSP is on the path of becoming an important plant protein source in the food and nutraceutical industries, due to its high nutritional and bioactive peptide development values, all of these advances were sourced from the various physiological functions of hydrolysates of HSP (6).

## Conclusion

The present study extracted the hemp seed storage proteins from the Longma 5 variety grown in North China with different methods, and then characterized them and analyzed the related genes. The results revealed that the modified Osborne method produced the best extraction of the hemp seed storage proteins. Edestins were the most abundant proteins in HSP, and 7S vicilin-like protein was the least. Edestins and albumin had a high ratio of Arg/Lys and high content of sulfur-containing amino acids, which indicated that they had a potential to promote cardiovascular health and serve as a rich thiol source to formulate highly nutritious foods. It also showed that amino acid composition of seed proteins extracted from different hemp cultivars was different, which could be a result of different genotypes. The knowledge of primary structure of these beneficial proteins is an important prerequisite for the development of foods with high nutritional value and identification of bioactive peptide fractions. The present research may, therefore, be viewed as a foundation for future research on the formulation of bioactive products and the development of healthier plant protein sources.

## Data Availability Statement

The original contributions presented in the study are included in the article/Supplementary Material, further inquiries can be directed to the corresponding author.

## Author Contributions

XS: conceptualization, methodology, validation, formal analysis, investigation, writing—original draft, and visualization. LW: supervision. QW and YL: resources. YS: formal analysis. All authors contributed to the article and approved the submitted version.

## Conflict of Interest

The authors declare that the research was conducted in the absence of any commercial or financial relationships that could be construed as a potential conflict of interest.

## Supplementary Material

Supplementary Figure 1 BLAST results of gene coding sequences.

Supplementary Figure 2 Secondary structure and 3D structure model predictions of hemp seed storage proteins. **(A)** Secondary predictions of the hemp seed storage proteins. **(B)** Tertiary structure prediction of the hemp seed storage proteins. All the results were predicted by phyre2.

Supplementary Table 1 Primer sequences for cloning genes encoding hemp seed storage proteins.
