# Supplementary figures and images for: Identification and Characterization of the Seed Storage Proteins and Related Genes of Cannabis sativa L
Source: Front Nutr. 2021 Jun 7;8:678421. doi: 10.3389/fnut.2021.678421 (PMC8215128; doi:10.3389/fnut.2021.678421)

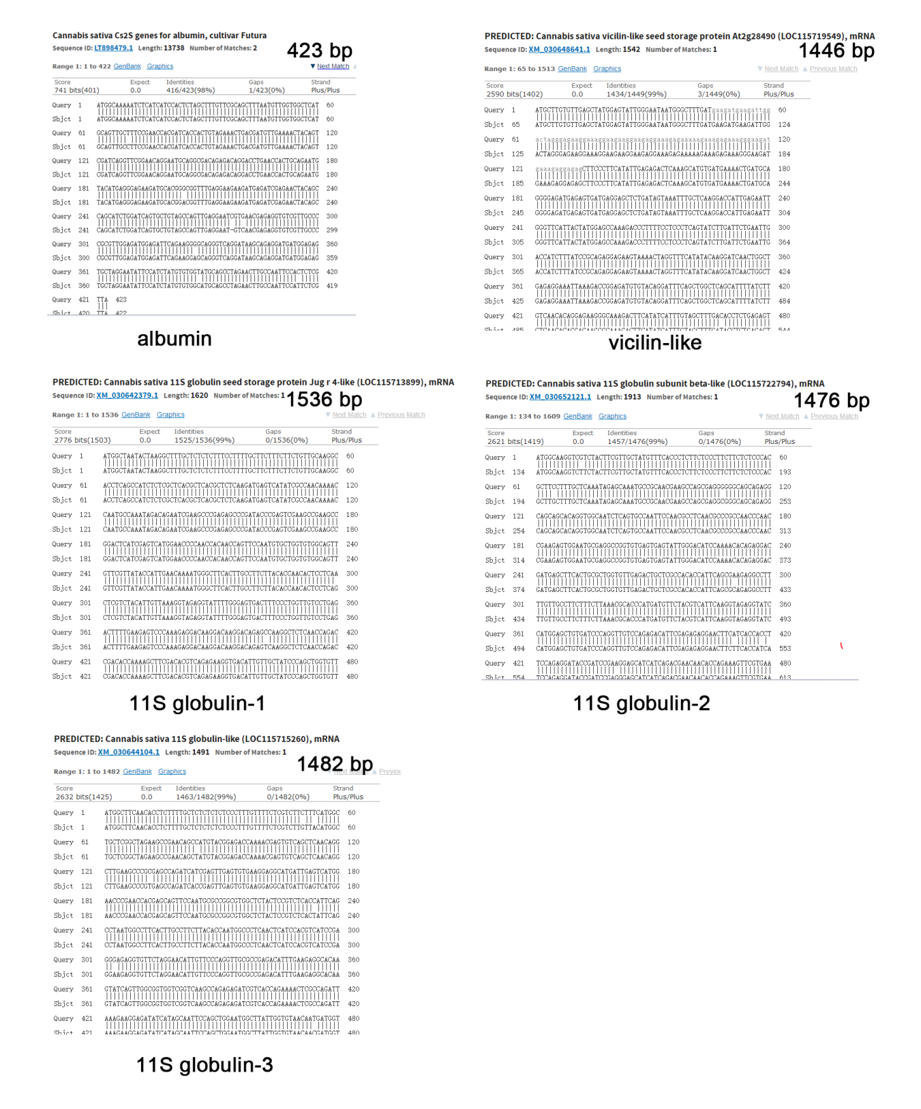

Supplement: Supplementary Figure 1 — BLAST results of gene coding sequences. [file Image_1.TIF]

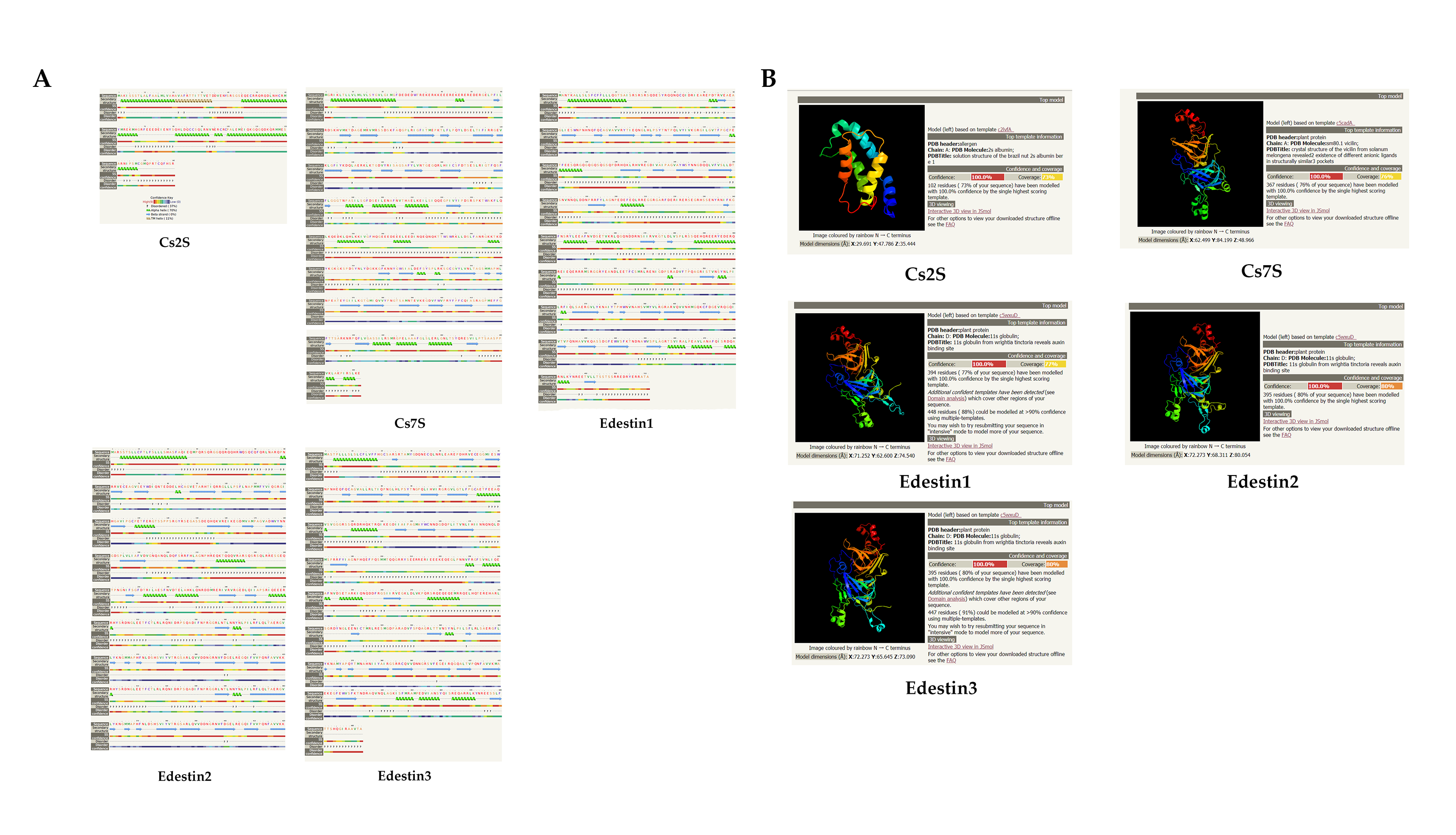

Supplement: Supplementary Figure 2 — Secondary structure and 3D structure model predictions of hemp seed storage proteins. (A) Secondary predictions of the hemp seed storage proteins. (B) Tertiary structure prediction of the hemp seed storage proteins. All the results were predicted by phyre2. [file Image_2.TIF]
